# Microbially reduced flavins destabilize the TiO_2_ passive film of titanium under bulk aerobic conditions

**DOI:** 10.1093/nsr/nwag426

**Published:** 2026-07-11

**Authors:** Jiaqi Li, Jia Lu, Yongqiang Fan, Feng Li, Xiangying Meng, Arjan Mol, Fuhui Wang, Tingyue Gu, Upadrasta Ramamurty, Dake Xu, Derek R Lovley

**Affiliations:** Electrobiomaterials Institute, Key Laboratory for Anisotropy and Texture of Materials (Ministry of Education), Northeastern University, Shenyang 110819, China; School of Materials Science and Engineering, Northeastern University, Shenyang 110819, China; State Key Laboratory of Digital Steel, School of Materials Science and Engineering, Northeastern University, Shenyang 110819, China; College of Life and Health Sciences, Northeastern University, Shenyang 110819, China; College of Life and Health Sciences, Northeastern University, Shenyang 110819, China; Key Laboratory of Systems Bioengineering (Ministry of Education), School of Chemical Engineering and Technology, SynBio Research Platform, Collaborative Innovation Center of Chemical Science and Engineering, Tianjin University, Tianjin 300072, China; Liaoning Academy of Materials, Shenyang 110167, China; Department of Materials Science and Engineering, Delft University of Technology, Delft 2628 CD, The Netherlands; Electrobiomaterials Institute, Key Laboratory for Anisotropy and Texture of Materials (Ministry of Education), Northeastern University, Shenyang 110819, China; School of Materials Science and Engineering, Northeastern University, Shenyang 110819, China; State Key Laboratory of Digital Steel, School of Materials Science and Engineering, Northeastern University, Shenyang 110819, China; Department of Chemical & Biomolecular Engineering, Institute for Corrosion and Multiphase Technology, Ohio University, Athens, OH 45701, USA; School of Mechanical and Aerospace Engineering, Nanyang Technological University, Singapore 639798, Singapore; Electrobiomaterials Institute, Key Laboratory for Anisotropy and Texture of Materials (Ministry of Education), Northeastern University, Shenyang 110819, China; School of Materials Science and Engineering, Northeastern University, Shenyang 110819, China; State Key Laboratory of Digital Steel, School of Materials Science and Engineering, Northeastern University, Shenyang 110819, China; Electrobiomaterials Institute, Key Laboratory for Anisotropy and Texture of Materials (Ministry of Education), Northeastern University, Shenyang 110819, China; School of Materials Science and Engineering, Northeastern University, Shenyang 110819, China; Department of Microbiology, University of Massachusetts Amherst, Amherst, MA 01003, USA

**Keywords:** microbial corrosion mechanism, passive film, metal reduction, marine microbe *shewanella algae*, electrobiocorrosion

## Abstract

Passive metals protected by surface oxide films dominate modern structural materials. Thus, understanding the mechanisms governing passive film breakdown is essential. The electronically insulating TiO_2_ passive film on titanium separates the underlying Ti^0^ from environmental oxidants, but microbial biofilms have been reported to catalyze titanium corrosion. Here we demonstrate that the common marine microbe *Shewanella algae* growing on titanium reduces TiO_2_ to destabilize the passive film. The biofilm consumes oxygen, generating anaerobic conditions near the metal interface even when the bulk environment remains aerobic. High-resolution characterizations reveal thinning of the passive layer and accumulation of lower-valence titanium oxides. Electrochemical analyses show a 6-fold increase in corrosion current from Ti^0^ accompanied by enhanced pitting. Mutant studies and other approaches indicate that extracellular flavins function as an intermediary electron carrier for *S. algae* TiO_2_ reduction. These results demonstrate how microbes create localized microenvironments that accelerate corrosion of otherwise highly corrosion-resistant metals.

## Teaser

Marine *Shewanella algae* biofilms accelerate titanium corrosion under bulk aerobic conditions by consuming oxygen and directly reducing the protective TiO_2_ layer, revealing an unrecognized bioelectrochemical corrosion pathway.

## INTRODUCTION

Titanium and its alloys are widely used in marine, industrial, and biomedical applications because of their exceptional corrosion resistance [1,2], which can be attributed to the spontaneous formation of a thin (typically 5–10 nm) passive film composed primarily of hydrous TiO_2_ [3–5]. The passive film physically separates the underlying Ti^0^ from external corrosive agents and its low electronic conductivity limits electron transfer from the underlying Ti^0^ to external oxidants such as dissolved oxygen or protons.

Despite this inherent stability, titanium corrosion has been observed in some service conditions, including seawater heat exchangers and other marine systems, where failure can pose significant safety and economic risks [6,7]. A growing body of evidence suggests that microbial activity plays a central role in these corrosion phenomena, as titanium corrosion has repeatedly been observed in the presence of diverse aerobic and anaerobic microorganisms [8–12]. Microbial secretion of organic acids may be one mechanism for disrupting the passive film [13,14]. Sulfide generated by sulfate-reducing microorganisms forms TiS_2_ within the passive film [15–17]. Several studies proposed that microbial extracellular activity could enable electron transfer from Ti^0^ [10,18], but did not explain how such an activity could access the Ti^0^ covered with an insulating TiO_2_ passive film.

Recent studies have demonstrated that the electroactive microbe *Geobacter sulfurreducens* can electrically degrade the passive film. It transfers electrons to TiO_2_ with outer-surface electron transfer proteins evolved for the reduction of iron oxides in soil, thinning the passive film and introducing defects of reduced titanium oxides that increase film conductivity [12]. These changes destabilize the passive film and increase the susceptibility of the metal to corrosion.

Strict anaerobes like *G. sulfurreducens* are unlikely to colonize titanium surfaces in most marine or industrial environments, which are typically oxic. However, some ‘facultative’ microbes that can grow both aerobically and anaerobically are also capable of extracellular electron transfer. Among these, *Shewanella* species are the best studied [19]. Unlike the *Geobacter* species, which rely on direct physical contact with solid extracellular electron acceptors, *Shewanella* species predominantly transfer electrons via soluble flavin electron shuttles that are excreted and then reduced at the cell surface by outer-membrane porin-cytochrome conduits, such as the MtrCAB system [19–21]. Under anaerobic conditions, the reduced flavins diffuse away from the cell surface and reduce iron oxides, regenerating the oxidized flavin, which can undergo multiple cycles of reduction and oxidation.

The redox potential of hydrous TiO_2_ (−0.091 V) is comparable to that of microbially reducible iron oxides [12], suggesting that reduced flavins might also serve as electron shuttles for the TiO_2_ reduction. If so, flavin-mediated electron transfer could destabilize the titanium passive film under anaerobic conditions. Actively metabolizing *Shewanella* biofilms have previously been shown to create an anoxic environment near the biofilm/metal interface during corrosion of ferrous metals [22].

These considerations led us to hypothesize that the microbial biofilms formed by facultative, flavin-secreting electroactive bacteria can locally eliminate oxygen and enable flavin-mediated reduction of the TiO_2_ passive film that, in turn, compromises its protective and electronic properties under bulk aerobic conditions. To ascertain this, we conducted this study with the marine microbe *Shewanella algae* wherein we combined materials characterization, electrochemical analysis, and genetic perturbations targeting flavin-mediated electron transfer, to determine whether microbial activity can reduce TiO_2_ in the passive film to accelerate titanium corrosion when the bulk environment is aerobic.

## RESULTS AND DISCUSSION

### *Shewanella algae* reduces the TiO_2_ passive film

**Figure 1.**
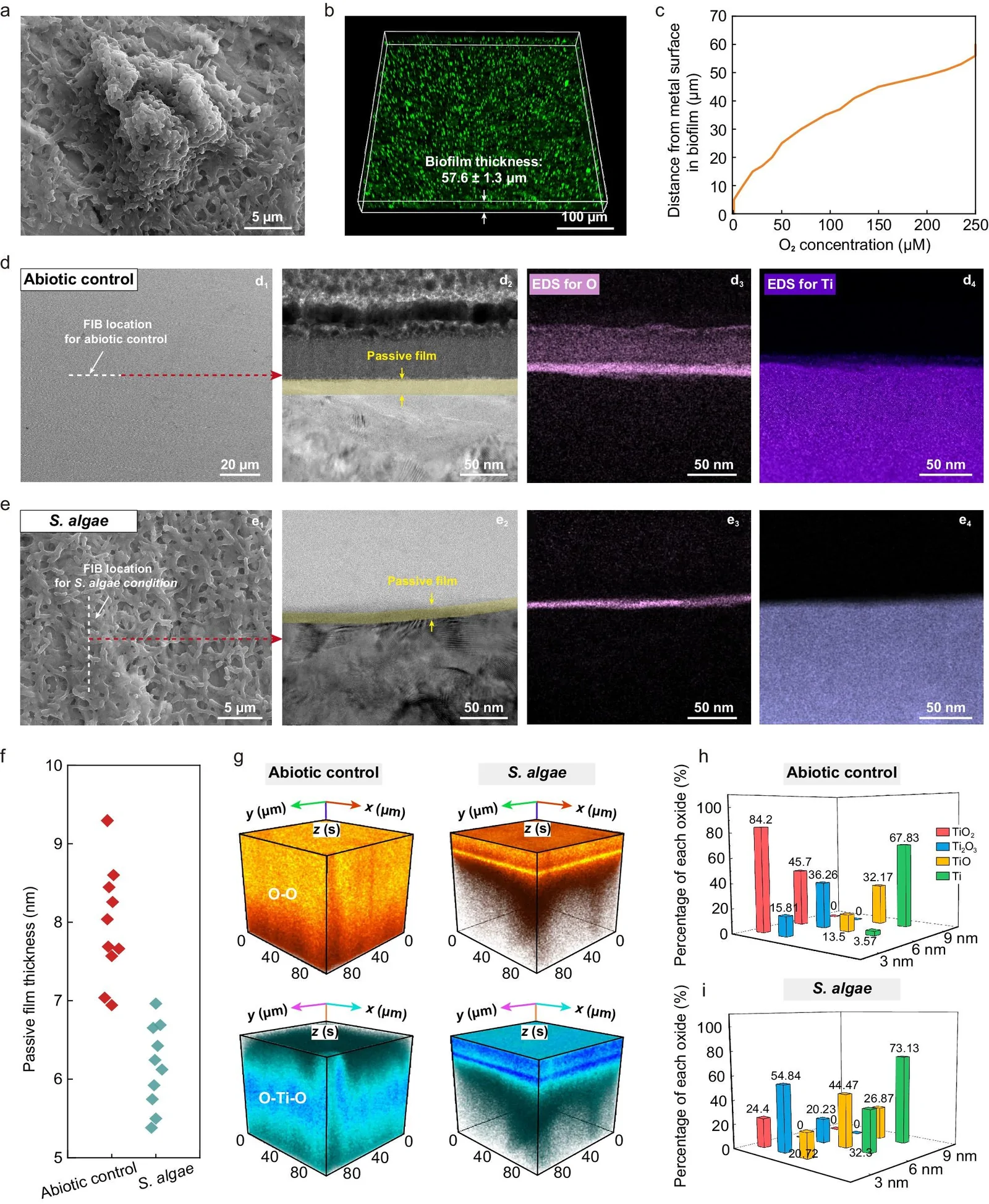
Effect of *S. algae* biofilm on titanium passive film. (a) Scanning electron micrograph and (b) confocal scanning laser micrograph after 14 days of incubation of *S. algae* and titanium. Biofilm observations at other incubation times and the medium pH value are provided in Fig. S1. (c) Depletion of dissolved oxygen with depth in *S. algae* biofilm. Cross-sectional characterization of the titanium passive film in (d) abiotic control medium and (e) incubation with *S. algae* after 21 days of incubation. Regions on the titanium surface identified with scanning electron microscopy (d_1_, e_1_) were prepared with focused ion beam (FIB) milling along the white dotted lines. High-resolution transmission electron microscopy (d_2_, e_2_) combined with energy-dispersive X-ray spectroscopy (d_3_, d_4_, e_3_, e_4_) revealed the passive film structure and the spatial distribution of titanium and oxygen. (f) Estimate of the titanium passive film thickness measured at ten independent locations from TEM images. (g) Three-dimensional distribution of O–O and Ti–O–Ti bonds from time-of-flight secondary ion mass spectrometry. Brighter colors indicate higher intensity, marking passive film boundaries. (h, i) Relative percentage of titanium oxides with depth from the surface as determined with X-ray photoelectron spectroscopy after 21 days of incubation (See Fig. S2 for survey spectra and high-resolution spectral scan data).

*Shewanella algae* grew as dense biofilms on the titanium surface (Fig. 1a, b), consuming oxygen to generate anaerobic conditions near the biofilm/metal interface (Fig. 1c). High-resolution transmission electron microscopy (TEM) and energy dispersive spectroscopy of titanium cross sections milled with focused ion beam (FIB) revealed substantial thinning of the titanium oxide passive film beneath *S. algae* biofilms (Fig. 1d–f). In the absence of *S. algae*, the passive film had a uniform thickness and straight boundaries (Fig. 1d), with an average thickness of 8.0 ± 0.7 nm (Fig. 1f). Under the *S. algae* biofilms, the passive film boundaries were irregular (Fig. 1e) and thinner with an average thickness of 5.9 ± 0.5 nm (Fig. 1f). Time-of-flight secondary ion mass spectrometry (TOF-SIMS) revealed a thinning and decrease in O–O bonds and an increase in Ti–O–Ti bonds at the metal surface, suggesting that TiO_2_ was reduced to lower titanium redox states (Fig. 1g). X-ray photoelectron spectroscopy (XPS) confirmed a loss of TiO_2_ in surface layers in the presence of *S. algae* (Fig. 1i) with a concomitant increase in lower-valence titanium species (Fig. 1h). Although each technique has inherent limitations, the multiple independent approaches, such as FIB-TEM, TOF-SIMS and XPS, all indicated that the TiO_2_ passive layer was reduced.

### Changes in electronic and mechanical properties associated with TiO_2_ reduction

**Figure 2.**
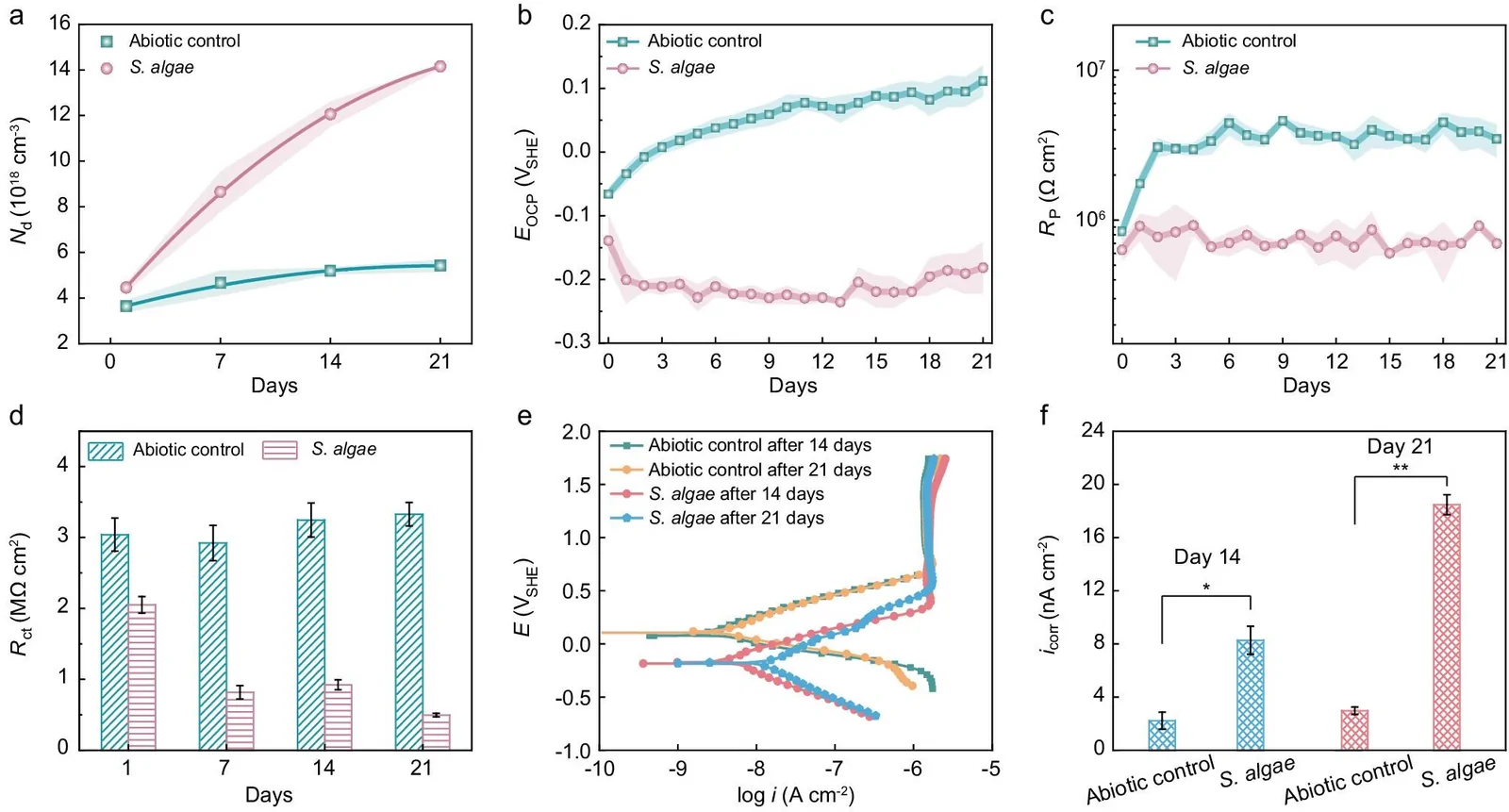
Electronic properties of the titanium sample associated with TiO_2_ reduction by *S. algae* biofilm. (a) Donor density (*N*_d_) from Mott-Schottky analysis after 21 days of incubation (Mott-Schottky plots in Fig. S3). (b) Open circuit potential (*E*_OCP_). (c) Linear polarization resistance (*R*_p_). (d) Charge-transfer resistance (*R*_ct_) determined from electrochemical impedance spectroscopy data fitting (Fig. S4 and Table S1). (e) Potentiodynamic polarization analysis and (f) corrosion current (*i*_corr_) determined from data in (e) (*P < 0.05, **P < 0.01). Results are the mean and standard deviation of three independent replicates (*n* = 3).

Multiple electrochemical analyses demonstrated that the reduction of TiO_2_ was associated with a substantial change in the electronic transfer properties of the titanium. The Mott-Schottky analysis revealed that during incubation of the titanium with *S. algae*, a marked increase in defect densities occurs (Fig. 2a), consistent with the increased accumulation of more reduced states of titanium within the surface layer of the metal [23]. The lower insulating capacity of the passive layer in the presence of *S. algae* is associated with a negative open circuit potential (OCP) (Fig. 2b) and lower polarization resistance (*R*_p_) (Fig. 2c) and charge-transfer resistance (*R*_ct_) (Fig. 2d), indicating that passive film degradation facilitates electrochemical reactions associated with titanium corrosion. This becomes further evident from potentiodynamic polarization analysis (Fig. 2e), which shows that after 21 days of incubation the corrosion current density (*i*_corr_) is 6-fold higher in the presence of *S. algae* than in the abiotic control (Fig. 2f).

**Figure 3.**
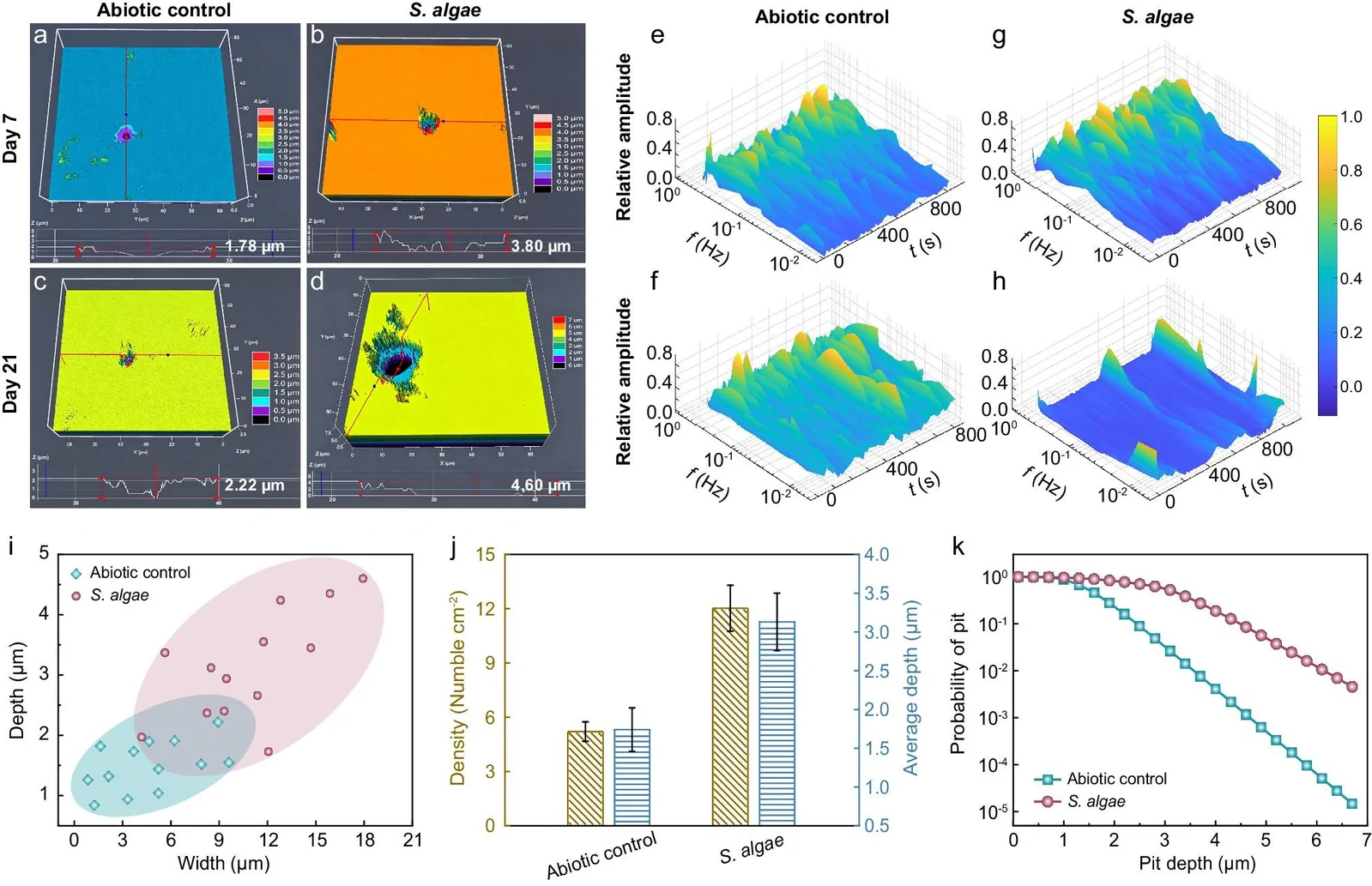
Analysis of titanium corrosion driven by *S. algae*-induced TiO_2_ reduction. (a–d) Confocal scanning laser micrographs of typical pits. (e–h) Hilbert spectra of electrochemical noise in the abiotic control and the *S. algae* inoculated medium (typical data in Fig. S5). (i) Distribution of pit depth-width profile derived from pits analysis in (a–d). (j) Pitting density (left axis, diagonal-patterned bars) and average pitting depth (right axis, horizontal-patterned bars). (k) Probability distribution of pit depths (probabilistic analysis and statistical analysis in Fig. S6).

As expected from the higher corrosion currents, incubation of the titanium with *S. algae* generated greater pitting as evidenced from visualization of the deepest pits (Fig. 3a–d), estimates of pit depth profile distribution (Fig. 3i), pitting density and average depth (Fig. 3j), and probability of maximum pitting depth (Fig. 3k). Corrosion events were further monitored in real time with the Hilbert spectral analysis of electrochemical noise (Fig. 3e–h). Early in the experiment (day 7), both conditions exhibited only short-lived transients, which is indicative of sub-stable pits that rapidly repassivate. By day 21, the abiotic control showed limited metastable pitting, whereas titanium exposed to *S. algae* displayed stronger, sustained transients, characteristic of stable localized pitting. These results further indicate that *S. algae* activity weakened the TiO_2_ passive film and promoted the transition from transient to stable pitting corrosion.

**Figure 4.**
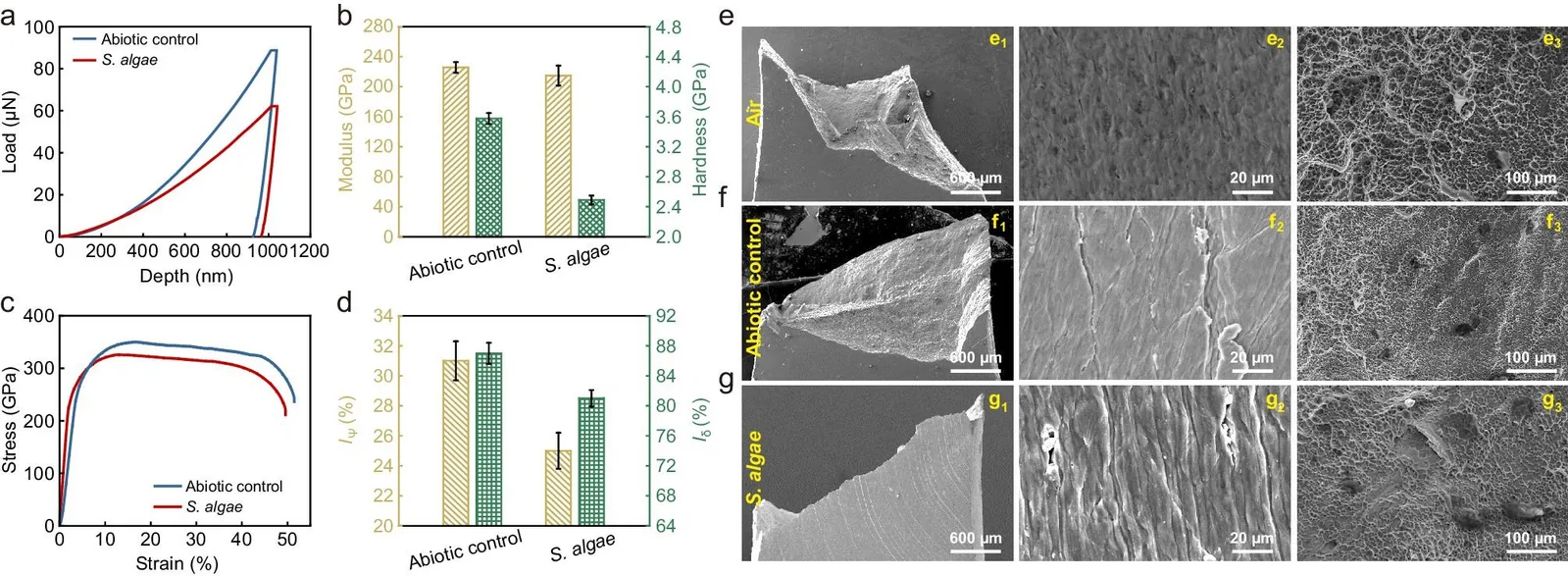
Mechanical properties of the titanium sample associated with TiO_2_ reduction by *S. algae* biofilm. (a) Depth-sensing indentation curves after 21 days of immersion. (b) Modulus (left axis, diagonal-patterned bars) and hardness (right axis, grid-patterned bars) values determined from data in (a). (c) Tensile stress–strain curves. (d) Stress corrosion cracking (SCC) susceptibility evaluated by area reduction loss *I_Ψ_* (left axis, diagonal-patterned bars) and elongation loss *I_δ_* (right axis, grid-patterned bars). (e–g) Morphology of fractured Ti samples: surface (e_1_, f_1_, g_1_), side fracture (e_2_, f_2_, g_2_), and front fracture (e_3_, f_3_, g_3_) observed in air (e), sterile medium (f), and *S. algae*-inoculated media (g), respectively. Results are the mean and standard deviation of three independent replicates (*n* = 3).

After 21 days of incubation in the presence of *S. algae*, the titanium hardness decreased significantly along with a slight decrease in the elastic modulus that was within the experimental variability (Fig. 4a, b). Tensile testing demonstrated a reduction in yield strength and decreases in both the elongation to failure and cross-sectional reduction (Fig. 4c, d), indicating reduced resistance to deformation and fracture during loading. Fracture surface analysis further revealed deeper side cracks and a higher density of microvoid features in samples exposed to *S. algae* compared to those tested with sterile controls (Fig. 4e and f). Taken together with the corrosion results, these observations are consistent with microbial activity promoting localized surface degradation and defect formation that can facilitate crack initiation and increase susceptibility to stress-corrosion-related damage, rather than indicating a substantial change in the intrinsic bulk mechanical properties of the titanium.

### Mechanism of the TiO_2_ reduction caused by *S. algae*

The *Shewanella* species primarily transfer electrons to iron oxides, a common electron acceptor in anaerobic soils and sediments, via excreted flavin electron shuttles that are reduced by the outer-surface cytochrome of the MtrCAB porin cytochrome conduit [19–21]. The midpoint redox potential for the reduction of the hydrous, poorly crystalline TiO_2_ in the passive film to Ti_2_O_3, hydr_ [24–27]:


(1) \begin{eqnarray*}
2{\mathrm{Ti}}{{\mathrm{O}}}_{2,{\mathrm{hydr}}} &+& 2{{\mathrm{H}}}^ + + 2{{\mathrm{e}}}^ - \Leftrightarrow {\mathrm{T}}{{\mathrm{i}}}_2{{\mathrm{O}}}_{3,{\mathrm{hydr}}}\\
&+& {{\mathrm{H}}}_2{\mathrm{O}}\ ({E}^0 = - 0.091\,{{\mathrm{V}}}_{{\mathrm{SHE}}})
\end{eqnarray*}

is more positive than for the oxidation of reduced riboflavin [28,29]:


(2) \begin{eqnarray*}
{\mathrm{R}}{{\mathrm{F}}}_{{\mathrm{OX}}} &+& 2{{\mathrm{H}}}^ + + 2{{\mathrm{e}}}^ - \Leftrightarrow {\mathrm{R}}{{\mathrm{F}}}_{{\mathrm{Red}}}\\
&&({E}^0 = - 0.22\,{\mathrm{\ }}{{\mathrm{V}}}_{{\mathrm{SHE}}}).
\end{eqnarray*}

The reduction of TiO_2_ leads to the lower-valence titanium oxides [24,30]:


(3) \begin{eqnarray*}
3{\mathrm{Ti}}{{\mathrm{O}}}_{2,{\mathrm{hydr}}} &+& 2{{\mathrm{H}}}^ + + 2{{\mathrm{e}}}^ - \Leftrightarrow {\mathrm{T}}{{\mathrm{i}}}_3{{\mathrm{O}}}_5\\
&+& {{\mathrm{H}}}_2{\mathrm{O}}\ ({E}^0 = 0.152\,{\mathrm{\ }}{{\mathrm{V}}}_{{\mathrm{SHE}}})
\end{eqnarray*}

and


(4) \begin{eqnarray*}
4{\mathrm{Ti}}{{\mathrm{O}}}_{2,{\mathrm{hydr}}} &+& 2{{\mathrm{H}}}^ + + 2{{\mathrm{e}}}^ - \Leftrightarrow {\mathrm{T}}{{\mathrm{i}}}_4{{\mathrm{O}}}_7\\
&+& {{\mathrm{H}}}_2{\mathrm{O}}\ ({E}^0 = 0.496\,{\mathrm{\ }}{{\mathrm{V}}}_{{\mathrm{SHE}}}).
\end{eqnarray*}

The hydrous Ti_3_O_5_ and Ti_4_O_7_ that result from Equations (3) and (4) are sub-stabilized and eventually transform to Ti_2_O_3_ in the passive film [31].

**Figure 5.**
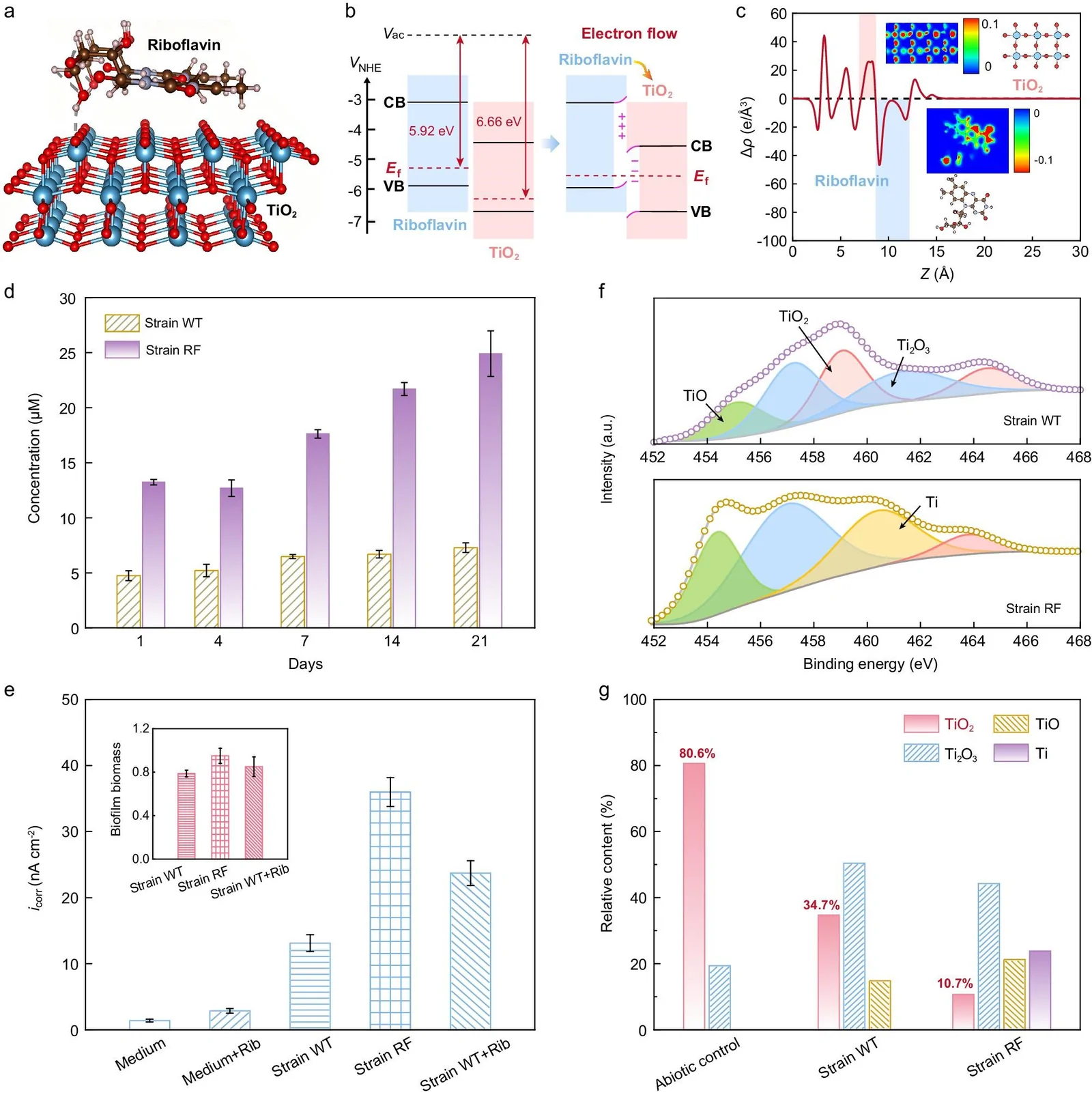
Electron transfer mechanism of *S. algae* driving TiO_2_ reduction. (a) Density functional theory calculated adsorption configuration illustrating heterojunction interfaces between riboflavin and TiO_2_. (b) Schematic illustration of the DFT-modeled charge transfer mechanism at the riboflavin/TiO_2_, with work functions and electron flow directions indicated (calculated potential parameters in Fig. S8). (c) Planar-averaged electron density difference Δ*ρ*(z) for riboflavin/TiO_2_, where positive and negative regions represent electron accumulation and depletion, respectively. (d) Riboflavin concentrations in media inoculated with the strain wild-type (WT) and strain riboflavin-overproducing (RF) *S. algae*. (e) The *i*_corr_ value after 21 days of anaerobic incubation in different conditions, derived from potentiodynamic polarization curves (Fig. S9). The inset displays the corresponding biofilm biomass for selected conditions. (f and g) X-ray photoelectron spectroscopy and relative percentage of titanium oxides at a sputtering depth of 3 nm after 21 days of anaerobic incubation with *S. algae* strain WT and RF, with lactate as the electron donor and the TiO_2_ of the passive film as the sole electron acceptor. The abiotic control data in panel (g) are taken from Fig. S2.

As expected, TiO_2_ abiotically oxidized the reduced riboflavin (Fig. S7). Density functional theory (DFT) modeling of the interface between riboflavin and TiO_2_ as a heterojunction [32,33] predicts a significant difference in the work functions of RF (5.92 eV) and TiO_2_ (6.66 eV) and suggests the electron transfer from reduced riboflavin to TiO_2_ based on Fermi level alignment (Fig. 5a–c). Differential charge density along the Z-direction (Fig. 5c) predicts electron depletion in riboflavin and accumulation on the TiO_2_ surface, suggesting a spontaneous electron transfer from riboflavin to TiO_2_ and providing atomic-scale justification of interface electron injection.

Extracellular riboflavin is present in wild-type *S. algae* cultures (Fig. 5d). Riboflavin concentrations are more than 2-fold higher in cultures of strain RF, a genetic construct designed to generate additional riboflavin (Fig. 5d). The addition of riboflavin had no impact on the corrosion currents of abiotic incubations of titanium, consistent with the lack of cells to generate reduced riboflavin as an electron donor for TiO_2_ reduction (Fig. 5e). However, amending wild-type cultures with riboflavin increased corrosion currents over unamended cultures and the corrosion currents for strain RF were even higher (Fig. 5e), despite comparable biofilm biomass (Fig. 5e), suggesting that riboflavin accelerated the reduction of TiO_2_, making the underlying Ti^0^ more susceptible to corrosion.

To further evaluate the potential for *S. algae* for reducing the TiO_2_ in the passive film, *S. alage* was incubated with titanium coupons under anaerobic conditions in a defined medium with lactate as the potential electron donor. XPS analysis shows a substantial loss of TiO_2_ and an increase of reduced titanium species at the surface of the titanium in the presence of *S. algae* (Fig. 5f, g). The TiO_2_ reduction is more extensive with strain RF.

**Figure 6.**
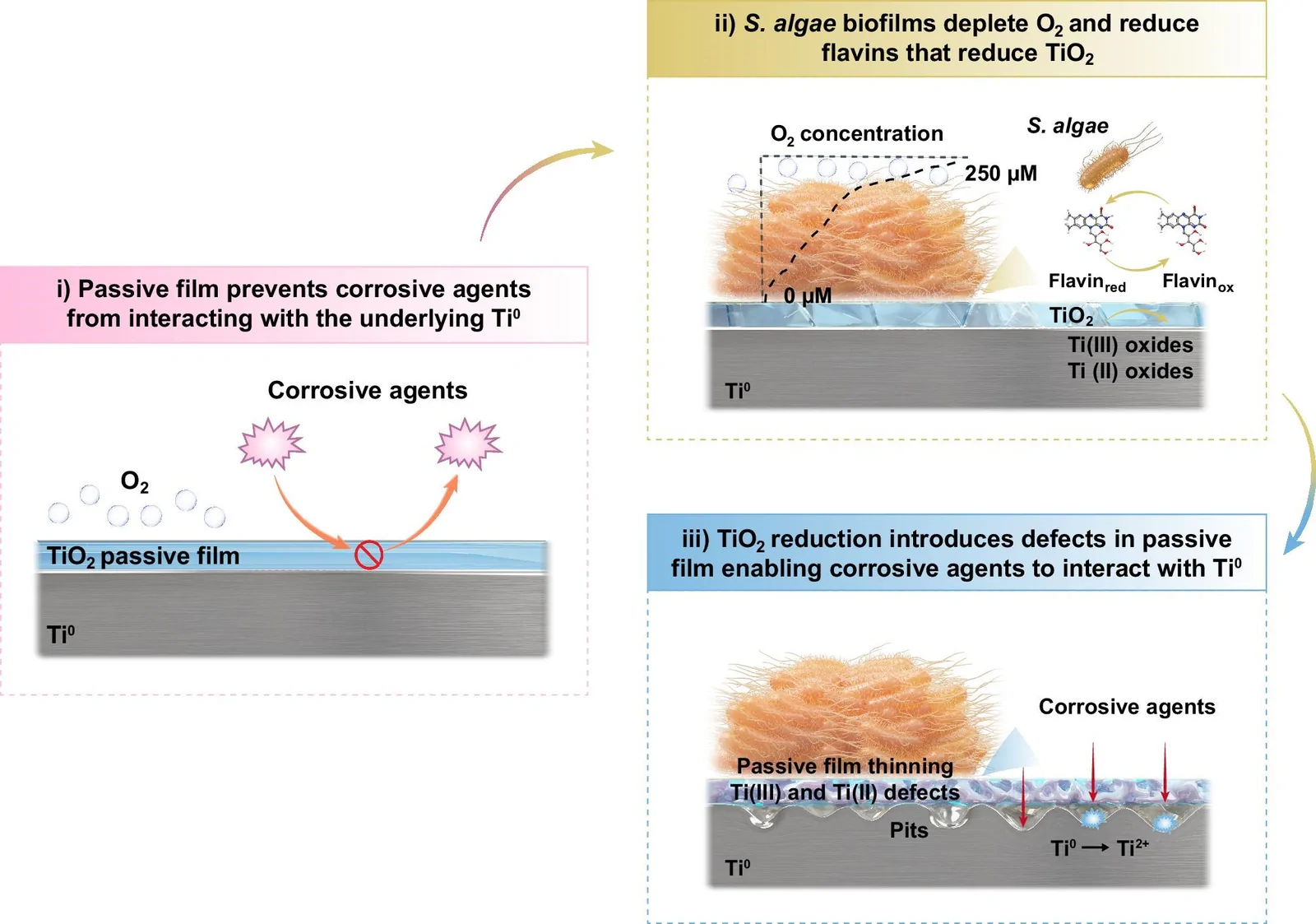
Model for *S. algae* modification of the titanium oxide passive film via anaerobic flavin-mediated reduction of TiO_2_ in a bulk aerobic environment.

Independently, each of the fine-scale surface analytical approaches can be expected to have possible limitations in interpretation, but collectively, the passive-film characterization, electrochemical measurements, genetic analyses, and riboflavin studies provide mutually supporting evidence that *S. algae* promotes TiO_2_ reduction within the passive film. The results are consistent with a model in which once *S. algae* establishes anaerobic conditions near the biofilm/metal interface and flavins function as an electron transfer shuttle to reduce and degrade the TiO_2_ passive film, making the underlying Ti^0^ more susceptible to corrosion (Fig. 6).

## CONCLUSION

The results demonstrate that, even in bulk aerobic environments, microbial flavins can reduce TiO_2_ within the titanium passive film, thinning the film and generating defect-rich reduced oxides that destabilize corrosion resistance. Many microbes have physiologies like *S. algae*; they also grow under both aerobic and anaerobic conditions and secrete flavin electron shuttles for the reduction of extracellular electron acceptors [19,34]. Thus, they are likely to generate similar corrosive-conducive microenvironments on titanium. The thinning may introduce breaches in the passive film where microbes and/or protons can directly contact the underlying Ti^0^ [12]. Furthermore, the decreased electrical resistance associated with the increased abundance of defects may permit electron transfer across the remaining intact titanium oxide film, analogous to the electron transfer to microbes across the intact chromium oxide-rich passive film of stainless steel [35].

Preventing biofilm formation would be the most direct strategy to protect titanium surfaces, but this is difficult in many practical environments [36,37]. The results suggest an alternative strategy: designing passive films that are intrinsically resistant to microbial reduction. Because flavin-mediated TiO_2_ reduction was the initiating step leading to passive-film destabilization and enhanced corrosion, future alloy-development efforts might focus on modifying passive-film composition, electronic structure, and defect chemistry to reduce susceptibility to microbial reduction. Recent advances in titanium-alloy design demonstrate that alloying can substantially alter passive-film stability and corrosion resistance [38,39], suggesting a promising route for mitigating microbiologically influenced corrosion in both marine and biomedical environments.

## MATERIALS AND METHODS

### Material, bacterial strains and biofilm characterization

Commercially pure titanium cylindrical rods (10 mm diameter, 3 mm height) were supplied by the Institute of Metal Research, Chinese Academy of Sciences (Shenyang, China). Specimens with a surface area of 0.785 cm^2^ were mounted in epoxy resin with a copper wire connection for electrochemical testing. The exposed surfaces were ground to 1000-grit using SiC paper and sterilized under UV irradiation for 30 min before use.

*Shewanella algae* (MCC 1A11468), obtained from the Marine Culture Collection of China, was cultivated in a 2216E culture medium. To investigate flavin-mediated extracellular electron transfer, a riboflavin-overproducing *S. algae* strain (denoted as strain RF) was constructed by heterologous overexpression of the *ribACDEH* operon from *Shewanella oneidensis* MR-1.

Biofilm morphologies were observed under field emission scanning electron microscopy (SEM, Ultra-Plus, Zeiss, Germany) and confocal laser scanning microscopy (C2 Plus, Nikon, Japan). Biofilm biomass was assessed via crystal violet staining. All measurements were performed in triplicate. Dissolved oxygen gradients within the biofilm were measured using a microsensor (OX-5, Unisense A/S, Denmark).

### Passive film characterization

Titanium passive films were characterized using FIB milling (Helios G4, Thermo Fisher Scientific, USA). Depth-resolved chemical distributions were obtained using TOF-SIMS 5 (IONTOF, Germany). Surface chemical states of titanium oxides were characterized by XPS (ESCALAB250, Thermo Fisher Scientific, USA) with Al Kα radiation. Spectra were calibrated using the C1s peak and analyzed with CasaXPS software (Casa Software Ltd., UK).

### Corrosion analysis

Electrochemical measurements were conducted in three-electrode glass cells using an electrochemical workstation (Gamry 600, Gamry Instruments, USA). OCP, electrochemical impedance spectroscopy, linear polarization resistance, Mott–Schottky analysis, and potentiodynamic polarization tests were performed. Electrochemical noise measurements were conducted for long-term corrosion monitoring and analyzed using the Hilbert–Huang transform. Corrosion pit morphology and depth distributions were evaluated by confocal microscopy and Gumbel extreme value analysis.

### Mechanical property analysis

The hardness and modulus of the passive film were examined using a nanoindentation system (TI 950 TriboIndenter, Hysitron Inc., USA). Stress corrosion cracking behavior was evaluated by slow strain rate testing under both sterile and bacterial immersion conditions, followed by SEM fracture analysis.

### Analytical techniques and density functional theory calculations

Riboflavin concentrations were determined with high-performance liquid chromatography (UltiMate 3000, Thermo Scientific, USA) using a C18 analytical column (4.6 × 150 mm, 5 μm). The pH of the culture medium was measured with a pH meter (FE20-134 FiveEasy Plus, Mettler Toledo, Switzerland).

First-principles DFT calculations were performed using the Vienna Ab-initio Simulation Package with the Perdew-Burke-Ernzerhof functional and projector-augmented wave potential [33,40]. Dispersion interactions were treated using the DFT-D3 (vdW) corrections.

Detailed Materials and Methods parameters are provided in the Supplementary Materials.

## Supplementary Material

nwag426_Supplemental_File
