# Supplementary material for: Microbially reduced flavins destabilize the TiO2 passive film of titanium under bulk aerobic conditions
Source: Natl Sci Rev. 2026 Jul 11;13(16):nwag426. doi: 10.1093/nsr/nwag426 (PMC13527873; doi:10.1093/nsr/nwag426)
Supplement: Supplementary file 1 [file nwag426_supplemental_file.pdf]

Supplementary materials

Microbially reduced flavins destabilize the TiO_2 passive film of titanium under bulk aerobic conditions

Table of contents

Supplementary Figures and Tables 2
Supplementary Materials and Methods 12
Material preparation and bacterial strains construction 12
Biofilm characterization 12
Passive film characterization 12
Electrochemical and corrosion measurements 13
Mechanical property analysis 13
Analytical techniques and density functional theory calculations 13
References 14

Supplementary Figures and Tables

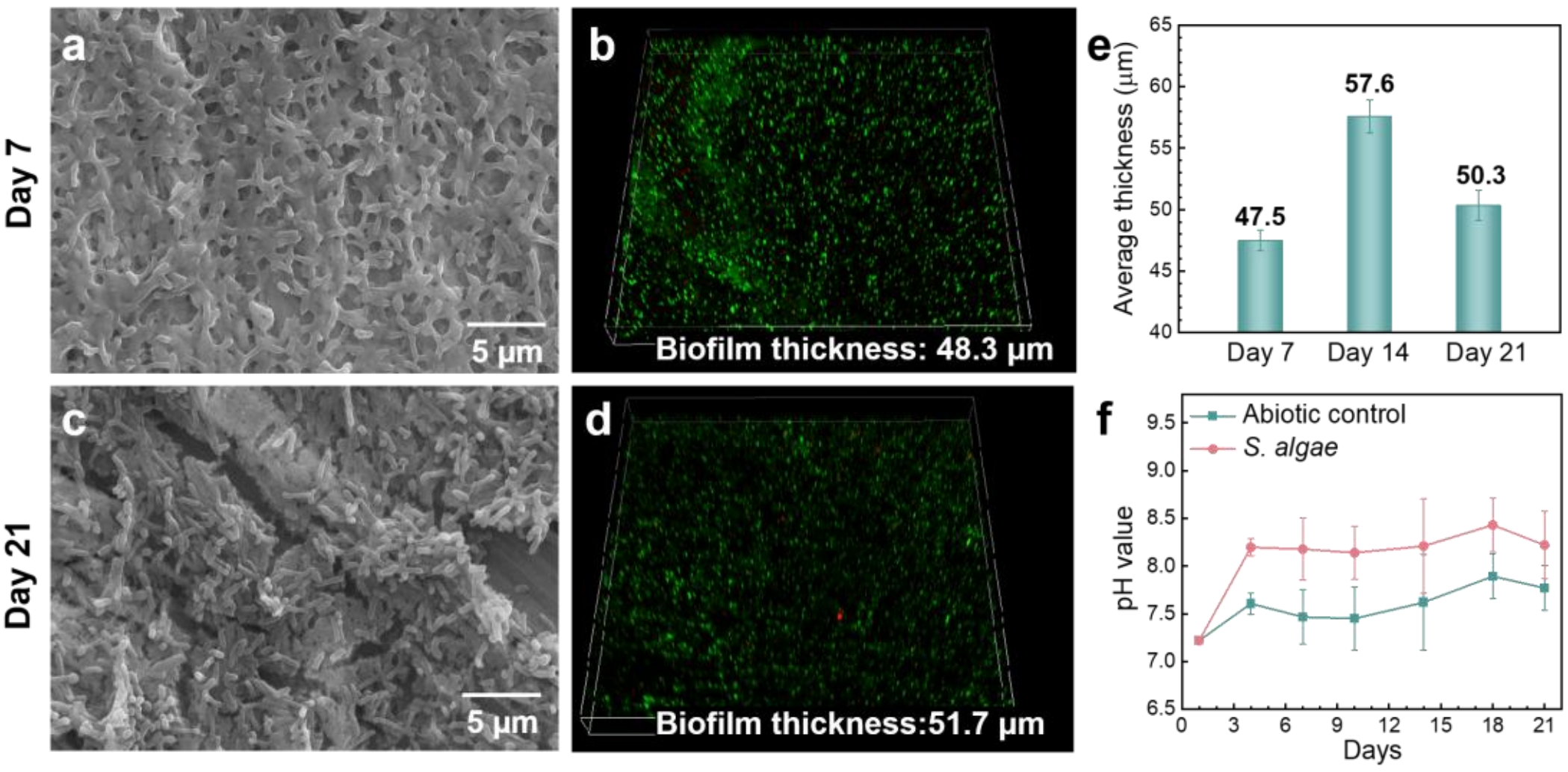

Fig. S1. (a)–(d) SEM and CLSM images of *S. algae* biofilms formed on Ti surfaces. (e) Average biofilm thickness measured from CLSM images. (f) pH values of the incubation media.

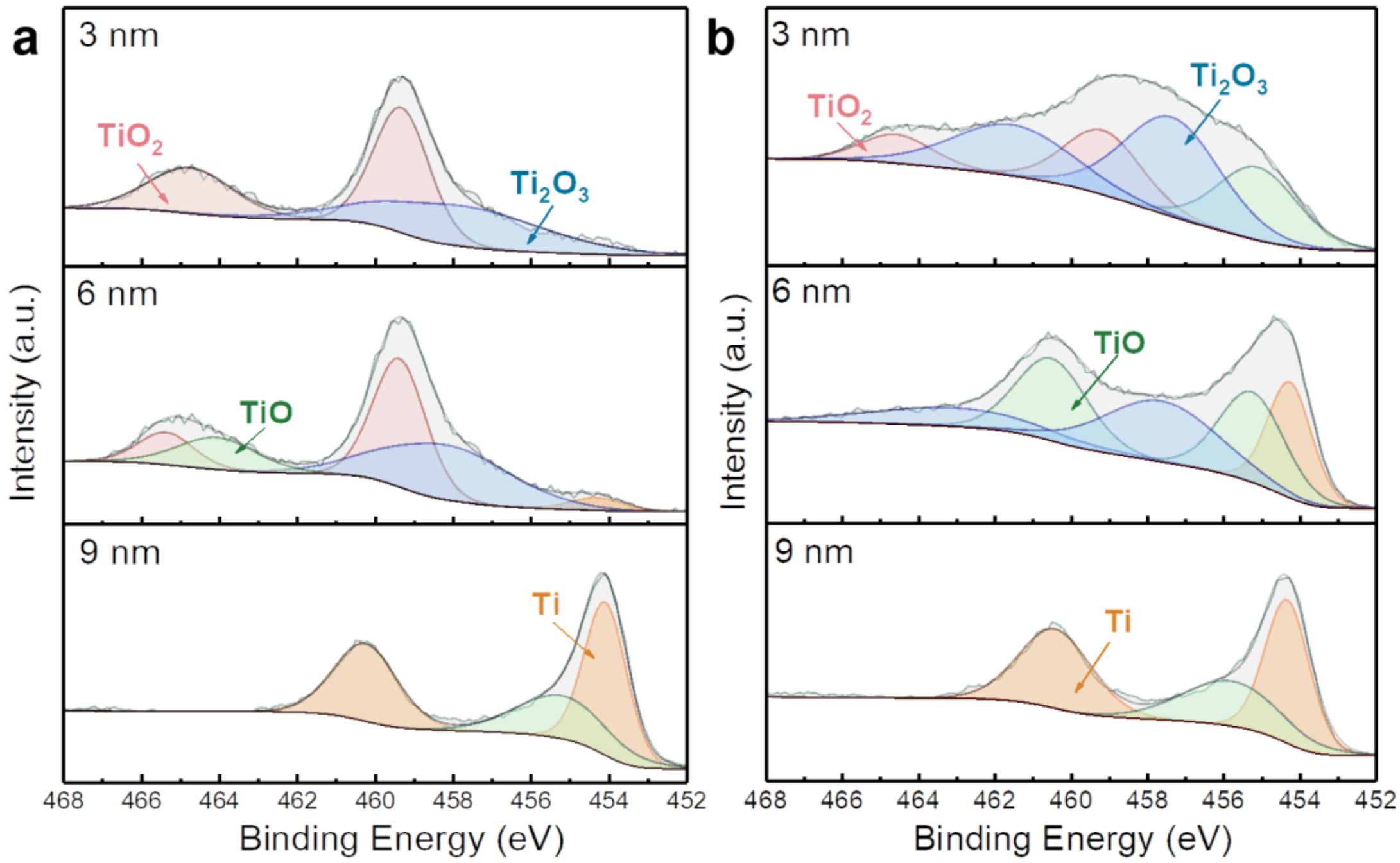

Fig. S2. High-resolution XPS spectra of Ti coupons after 21 days of incubation under (a) abiotic control conditions and (b) *S. algae*-inoculated conditions.

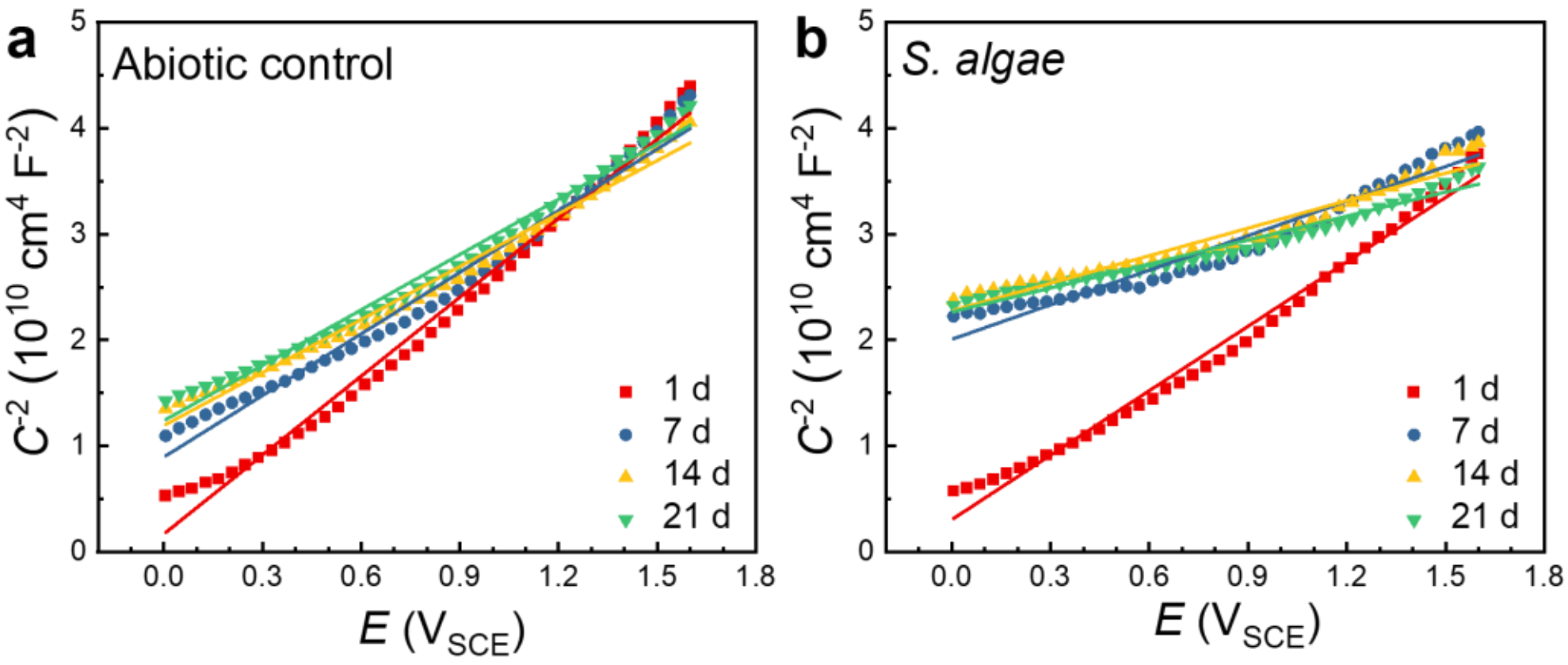

Fig. S3. Mott-Schottky plots obtained under (a) abiotic control conditions and (b) in the presence of *S. algae*. The positive slopes observed under both conditions indicate that the passive films exhibit n-type semiconducting behavior.

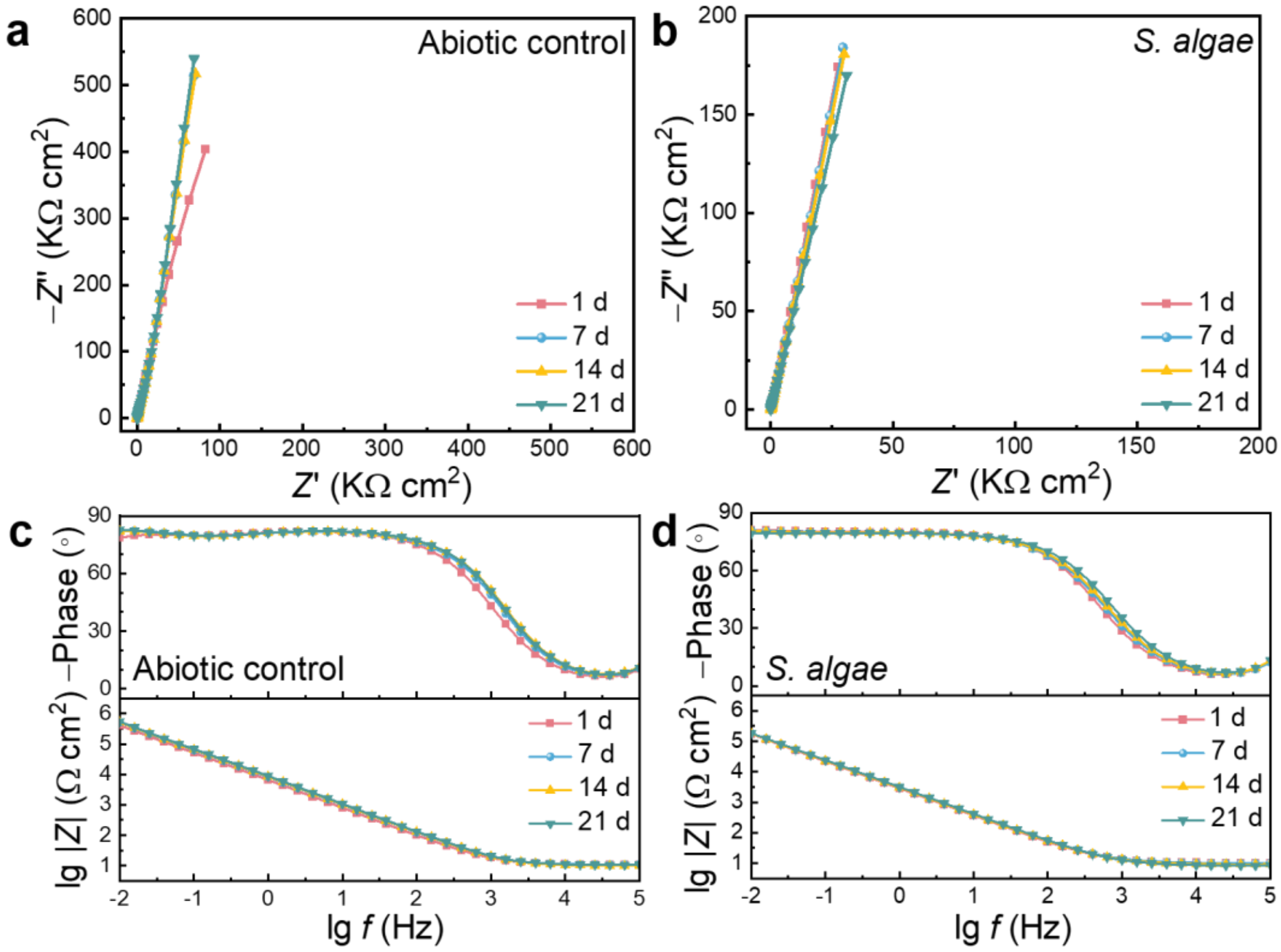

Fig. S4. Nyquist and Bode plots of Ti coupons after 21 days of immersion: (a, c) abiotic control and (b, d) *S. algae*-inoculated medium.

Table S1. EIS parameters were fitted using an *R(QR)(QR)* equivalent circuit. Values are reported as mean ± standard deviation from three independent experiments. The equivalent circuit model consists of the solution resistance (R_s), the passive film resistance (R_f), charge transfer resistance (R_{ct}), and two constant phase elements (Q_b and Q_{dl}). R_{ct} is a key parameter for evaluating corrosion resistance, with its reciprocal being proportional to the corrosion rate.

Duration (days)	R_S (×10^{-3}) (Ω cm^2)	Q_{dl} (μF cm^{-2} S^n)	n_{dl}	R_{ct} (MΩ cm^2)	Q_f (μF cm^{-2} s^n)	n_f	R_b (Ω cm^2)
**Ti in the abiotic medium**							
1	7.7 ± 2.6	34.8 ± 3.9	0.9 ± 0.01	3.0 ± 0.2	0.4 ± 0.07	0.8 ± 0.07	12.3 ± 1.5
7	7.6 ± 1.3	19.1 ± 0.1	0.9 ± 0.01	2.9 ± 0.3	0.6 ± 0.01	1.0 ± 0.01	9.2 ± 0.3
14	7.8 ± 1.2	24.9 ± 7.1	0.9 ± 0.01	3.3 ± 0.2	0.4 ± 0.2	0.7 ± 0.1	15.6 ± 2.4
21	16.7 ± 2.3	24.7 ± 3.4	0.9 ± 0.01	3.3 ± 0.2	0.3 ± 0.07	1.0 ± 0.04	10.6 ± 0.06
**Ti in the presence of *S. algae***							
1	18.1 ±1.4	65.0 ± 2.9	0.9 ± 0.01	2.1 ± 0.1	1.5 ± 0.1	0.7 ± 0.07	10.3 ± 0.3
7	24.5 ± 3.7	64.1 ± 0.5	0.9 ± 0.02	0.8 ± 0.1	0.9 ± 0.2	0.8 ± 0.1	10.4 ± 0.3
14	25.8 ± 3.7	65.9 ± 1.5	0.9 ± 0.04	0.9 ± 0.1	1.0 ± 0.2	0.8 ± 0.1	9.4 ± 0.3
21	29.8 ± 4.4	66.7 ± 4.5	0.9 ± 0.01	0.5 ± 0.02	1.6 ± 0.2	0.6 ± 0.1	9.9 ± 0.4

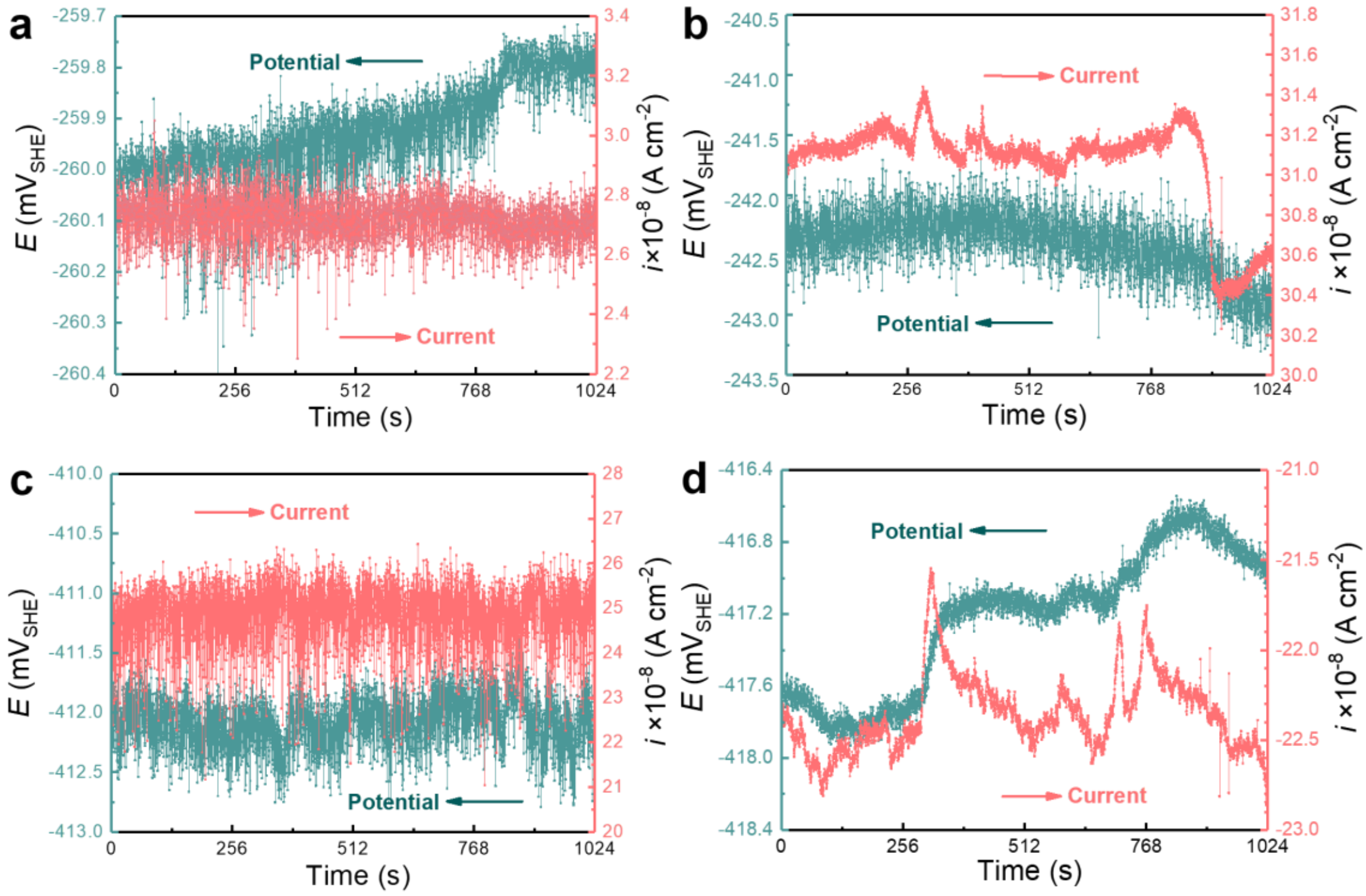

Fig. S5. Representative electrochemical noise data of Ti coupons: **(a)** day 7 and **(b)** day 21 in the abiotic control; **(c)** day 7 and **(d)** day 21 in *S. algae*-inoculated media.

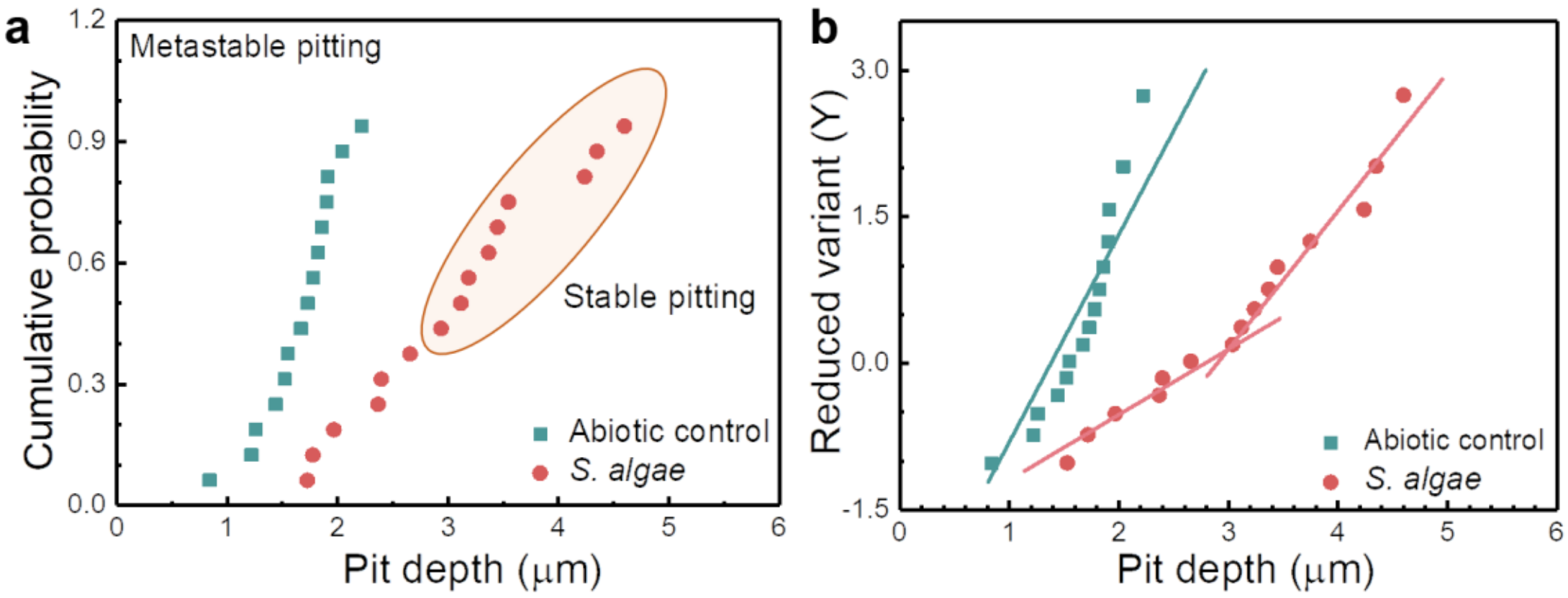

c

Medium	Metastable pit		Stable pit	
	α	μ	α	μ
Abiotic	0.48	1.36	---	---
S. algae	1.48	2.78	0.71	2.89

Fig. S6. Probabilistic analysis of the deepest corrosion pits in the abiotic control and the *S. algae* inoculated media after 21 days. (a) Statistical analysis of the cumulative probability F(*Y*) related to pit depths. The distribution of F(*Y*) of coupons in the presence of *S. algae* was broader (2.73–4.60 μm) than that in the sterile medium (0.84–2.22 μm). (b) Relationship between the reduced variant *Y*(−ln(−ln*F*)). Two fitting curves were observed for coupons exposed to bacteria, while only one curve appeared for coupons in the sterile medium. This suggests that an alternative mechanism for the formation of larger pits corresponds to the transition from metastable to stable pitting. The pit depth associated with this transition (~3 μm) is marked in (a). (c) Scale parameter α and location parameter μ obtained by linear fitting from panel (b).

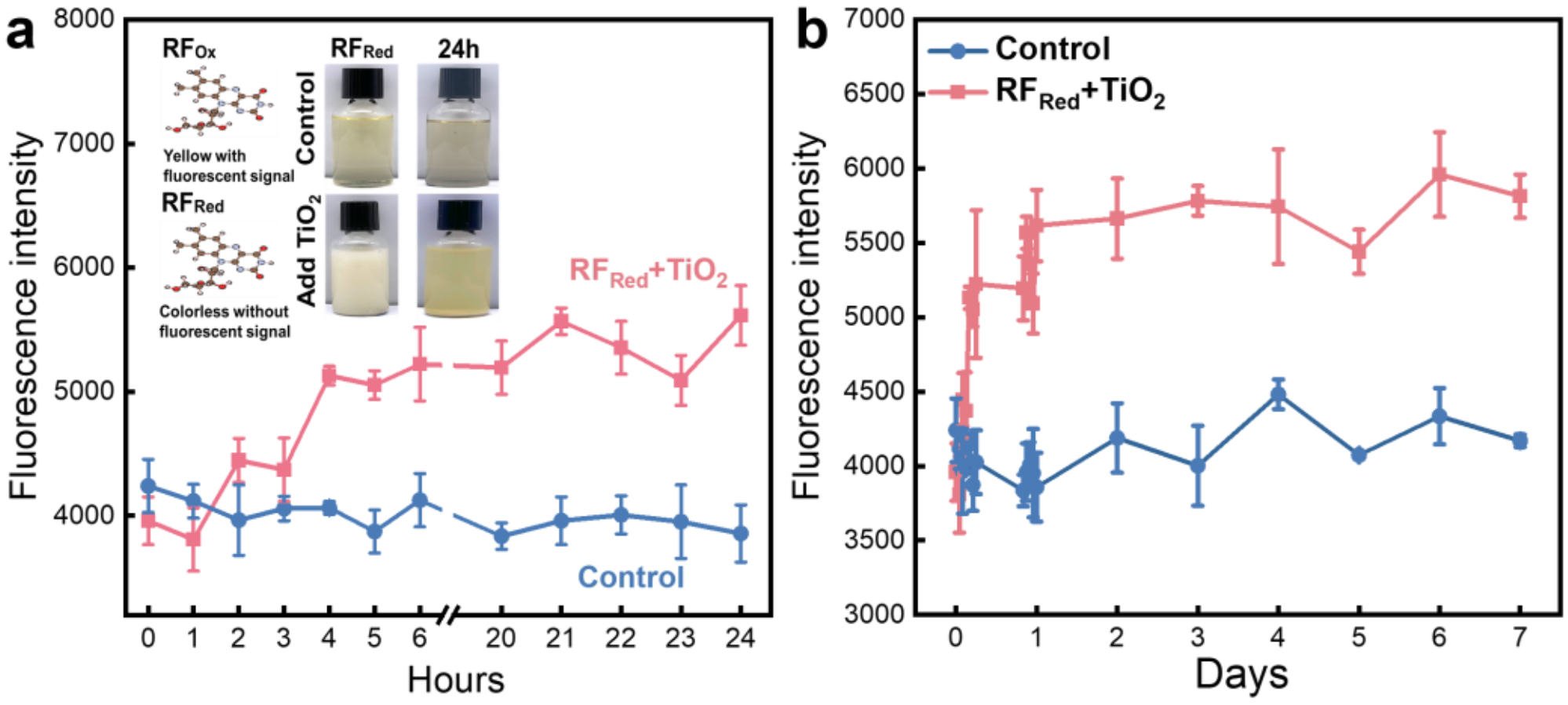

Fig. S7. Fluorescence intensity evolution of reduced riboflavin (RF_{Red}) after addition of TiO_2 (a) within 24 hours and (b) over 7 days.

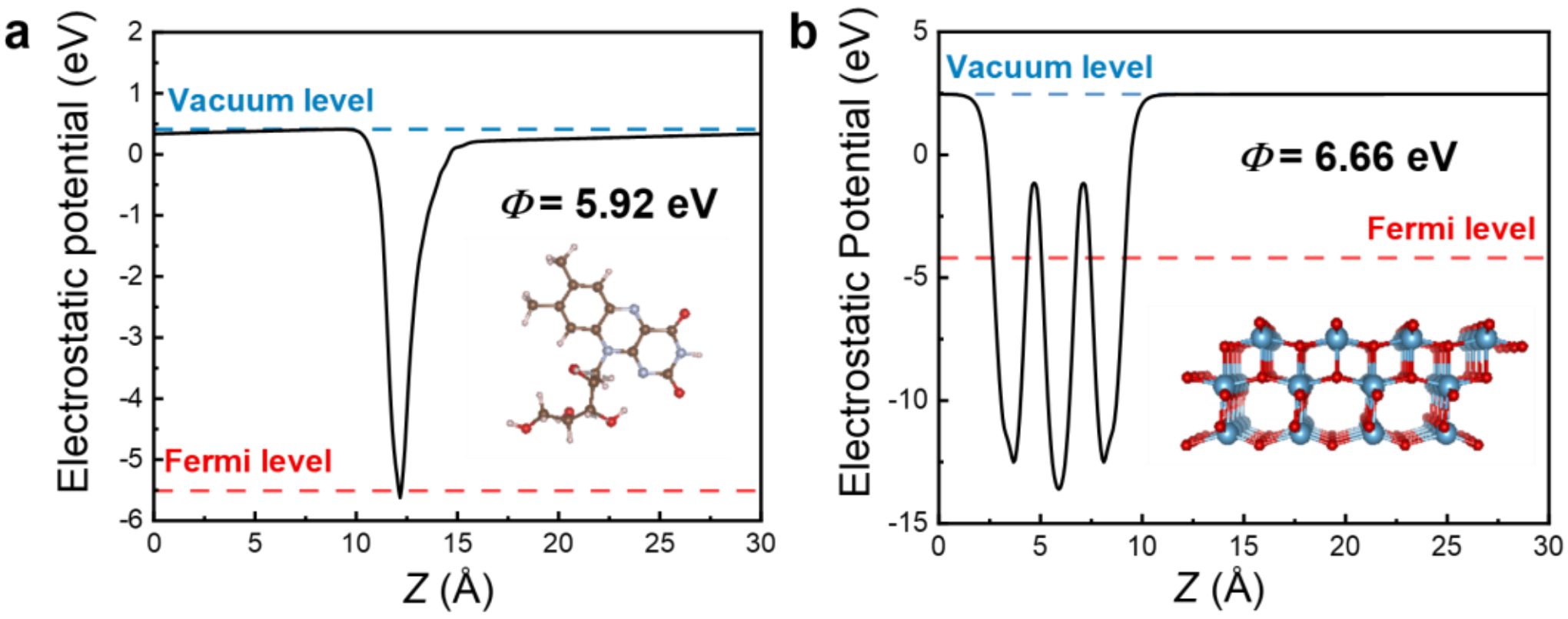

c

Potential (eV)	E_{CB}	E_{VB}	V_{ac}	E_f	Φ
RF	-3.41	-5.51	0.41	-5.51	5.92
TiO_2	-2.03	-4.20	2.47	-4.19	6.66

Fig. S8. Electrostatic potentials and work function (*Φ*) of (a) riboflavin and (b) TiO_2, where insets show the corresponding structural models. (c) Calculated potential parameters.

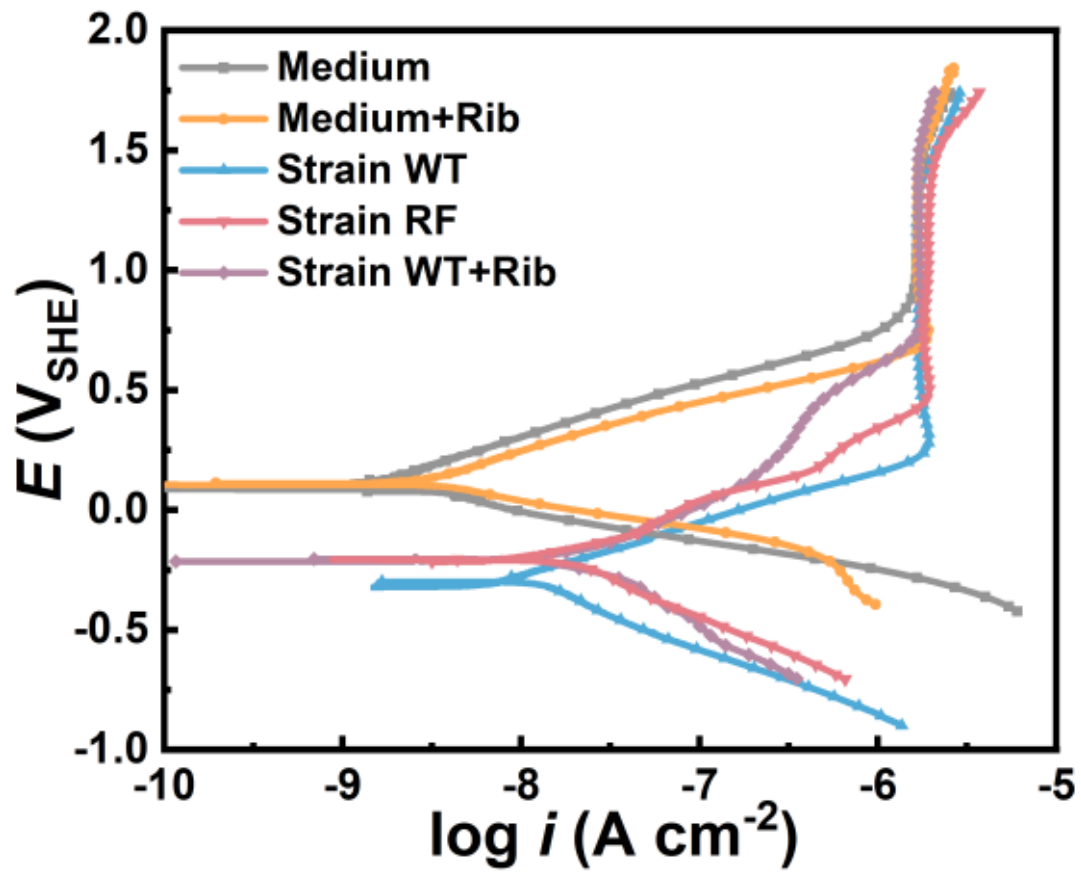

Fig. S9. Potentiodynamic polarization curves after 21 days of anaerobic incubation in different conditions.

Supplementary Materials and Methods

Material preparation and bacterial strain construction

The elemental composition (wt.%) was 0.01 Cu, 0.02 Fe, 0.01 C, 0.002 N, 0.10 O, 0.001 H, and balance Ti. Before experiments, specimens were ultrasonically cleaned with ethanol and rinsed with deionized water.

The 2216E medium was adjusted to pH 7.2 ± 0.2 and sterilized at 121 °C for 20 min. For the construction of the riboflavin-overproducing strain, the *ribA*, *ribD*, *ribC*, *ribE*, and *ribH* genes from *Shewanella oneidensis* MR-1 were assembled into the pYYDT plasmid and introduced into wild-type *S. algae* through conjugative transfer. For CRISPRi repression of the *Mtr* pathway, sgRNAs targeting *mtrA*, *mtrB*, and *mtrC* were co-expressed with catalytically inactive Cas9 under LacI regulation control, and the resulting system was introduced into *S. algae* via conjugation, resulting in transcriptional repression of the outer-membrane cytochrome conduit.

To investigate the potential for TiO_2 reduction, *S. alage* was incubated with titanium coupons in simulated seawater (per liter: 23.5 g NaCl, 0.7 g KCl, 10.6 g $MgCl_2 \cdot 6H_2O$, 1.1 g $CaCl_2$, Na_2HPO_4 1.1g, NaH_2PO_4 0.1g). The culture media were deoxygenated with filter-sterilized N_2 gas bubbling at least 30 min prior to use, establishing an anaerobic system for mechanistic validation under electron donor–sufficient conditions.

Biofilm characterization

For SEM observations, biofilms were fixed with glutaraldehyde and dehydrated through graded ethanol solutions [1]. Biofilm's viability was evaluated using the Live/Dead BacLight kit, where live and dead cells were visualized by green (488 nm) and red (559 nm) fluorescence, respectively. Biofilm thickness was reconstructed using NIS-Elements software (Nikon, Japan). Crystal violet absorbance was measured at 570 nm using a Synergy HTX microplate reader (BioTek, USA). All measurements were performed in triplicate.

Dissolved oxygen gradients within the biofilm were measured using a microsensor (OX-5, Unisense A/S, Denmark) with a tip diameter of approximately 5 μm. The probe was inserted stepwise from the bulk medium to the metal-biofilm interface, and data were analyzed with SensorTrace Profiling software (Unisense A/S, Denmark).

Passive film characterization

Prior to focused ion beam milling, a thin platinum layer was deposited onto specimen surfaces. Cross-sectional morphology was further characterized by TEM operated at 200 kV, and elemental distribution and film thickness were obtained with energy-dispersive X-ray spectroscopy (EDS). TOF-SIMS measurements were employed with a 250 eV Cs^+ sputtering beam over a 100 μm × 100 μm area. Although TOF-SIMS provides high sensitivity to surface chemical environments, measurements were conducted under identical sputtering and acquisition conditions for both biotic and abiotic samples, allowing the data to be used for assessing relative differences between the two experimental conditions.

XPS Spectra were calibrated using the C1s peak, and analyzed with CasaXPS software (Casa Software Ltd., UK). Given the potential uncertainties associated with peak deconvolution, charging effects, and Ar-ion sputtering-induced reduction, all

spectra were processed using identical fitting procedures and background settings, and XPS results were used for comparative analysis between biotic and abiotic conditions.

Electrochemical and corrosion measurements

All electrochemical tests used a saturated calomel electrode (SCE) as the reference electrode. Samples were allowed to stabilize at the open circuit potential (OCP) for 30 min prior to the measurements. Semiconductor properties of the passive film were evaluated using the Mott–Schottky analysis over −0.6-1.6 V range versus OCP with a 5 mV signal at 1 kHz [2, 3]. The donor density (N_d) was calculated from the slope of the linear region.

Linear polarization resistance (LPR) measurements were performed from −5 to +5 mV versus OCP at a scan rate of 0.125 mV s^{-1} to minimize perturbation of the electrode surface due to repeated measurements during immersion. Electrochemical impedance spectroscopy (EIS) measurements were carried out over 100 kHz to 0.01 Hz with a 5 mV perturbation. Potentiodynamic polarization curves were obtained by scanning the anodic and cathodic branches separately at 0.33 mV s^{-1} from OCP to characterize the overall electrochemical behavior. The corrosion current density (i_{corr}) was obtained from polarization curves by applying the Tafel extrapolation to the cathodic branch. All electrochemical measurements were repeated at least three times independently to ensure reproducibility.

Electrochemical noise (EN) measurements were performed within a Faraday cage with an electrochemical workstation (Zahner IM6, Zahner, Germany). Two identical titanium electrodes were used as working electrodes, while an SCE electrode served as the reference. Current fluctuations were recorded continuously for 504 h with a sampling interval of 0.25 s and analyzed using the Hilbert–Huang transform implemented in MATLAB procedure [4, 5].

After incubation, biofilms and corrosion products were removed following the ASTM standard G1-03 [6, 7]. Pit morphologies and depths were measured using confocal laser scanning microscopy operating in the bright field mode. The cumulative probability distribution of maximum pit depths was evaluated using the Gumbel extreme value distribution [1, 8].

Mechanical property analysis

Nanoindentation measurements employed a Berkovich indenter tip (radius = 50 nm) with a loading rate of 50 μN/s. Stress corrosion cracking behavior was evaluated by slow strain rate testing under both sterile and bacterial immersion conditions, with a constant strain rate of 5×10^{-7} /s. Fracture morphologies of the tested specimens were subsequently examined by SEM. Susceptibility to stress corrosion cracking was quantified based on the elongation loss ratio (I_δ) and reduction-in-area loss (I_ψ), determined by comparing specimens tested in immersion media with those tested in an inert atmosphere at room temperature [9-11].

Analytical techniques and density functional theory calculations

For HPLC analysis, the mobile phase consisted of methanol and 0.05 M ammonium acetate aqueous solution (85:15, v/v) at a flow rate of 1 mL min^{-1}. Riboflavin was detected with fluorescence measurements at an excitation wavelength of 460 nm and an emission wavelength of 520 nm (Synergy HTX, BioTek, USA).

In DFT calculations, a plane wave kinetic cutoff energy of 500 eV was applied. Dispersion interactions were treated using the DFT-D3 (vdW) corrections. Brillouin zone sampling was performed using the Γ-centered 2 × 2 × 1 and 4 × 4 × 1 k-mesh. Energy convergence criteria were set as 10^{-5} and 10^{-7} eV, and the Hellmann-Feynman force was 0.02 eV/Å.

References

1. Li J, Jin Y, Li J *et al.* The effect of dissolved oxygen and *Shewanella algae* on the corrosion mechanism of titanium in a simulated marine environment. *Corrosion Science*. 2024; **239**: 112400.
2. Di Paola A. Semiconducting properties of passive films on stainless steels. *Electrochimica Acta*. 1989; 34(2): 203-210.
3. Li X, Wang L, Fan L et al. Effect of temperature and dissolved oxygen on the passivation behavior of Ti-6Al-3Nb-2Zr-1Mo alloy in artificial seawater. *Journal of Materials Research and Technology*. 2022; 17: 374-391.
4. Meng G, Wei L, Tao Z *et al.* Effect of microcrystallization on pitting behavior of pure aluminum. *Corrosion Science*. 2009; **51**(9): 2151-2157.
5. Homborg AM, Tinga T, Westing EV *et al.* A Critical Appraisal of the Interpretation of Electrochemical Noise for Corrosion Studies. *Corrosion-Houston Tx*. 2014; **70**(10): 971-987.
6. Bearinger JP, Orme CA, Gilbert JL. In situ imaging and impedance measurements of titanium surfaces using AFM and SPIS. *Biomaterials*. 2003; **24**(11): 1837-1852.
7. Standard Practice for Preparing, Cleaning, and Evaluating Corrosion Test Specimens. *ASTM G1-03*. 2017.
8. Gholami M, Hoseinpoor M, Moayed MH. A statistical study on the effect of annealing temperature on pitting corrosion resistance of 2205 duplex stainless steel. *Corrosion Science*. 2015; **94**: 156-164.
9. Qi W, Gao Q, Zhao Y *et al.* Insight into the stress corrosion cracking of HP-13Cr stainless steel in the aggressive geothermal environment. *Corrosion Science*. 2021; **190**: 109699.
10. Qin Z, Pang X, Yan Y *et al.* Passive film-induced stress and mechanical properties of α-Ti in methanol solution. *Corrosion Science*. 2014; **78**: 287-292.
11. Beck TR, Blackburn MJ. Stress corrosion cracking of titanium alloys. *AIAA Journal*. 1967; **6**(2): 179.
